# Risk assessment and exposure to levels of naturally occurring aflatoxins in some packaged cereals and cereal based foods consumed in Accra, Ghana

**DOI:** 10.1016/j.toxrep.2018.11.012

**Published:** 2018-11-29

**Authors:** Nii Korley Kortei, Akwasi Akomeah Agyekum, Felicia Akuamoa, Vincent Kyei Baffour, Huseini Wiisibie Alidu

**Affiliations:** a Department of Nutrition and Dietetics, School of Allied Health Sciences, University of Health and Allied Sciences, PMB 31, Ho, Ghana; b Applied Radiation Biology Centre, Ghana Atomic Energy Commission, P. O. Box AE 1, Atomic, Accra, Ghana; c Toxicology Unit, Department of Chemistry, Council for Scientific and Industrial Research- Food Research Institute, P.O. Box M20, Accra, Ghana; d Department of Medical Laboratory Sciences, School of Allied Health Sciences, University of Health and Allied Sciences, PMB 31, Ho, Ghana

**Keywords:** Aflatoxins, rice, cereal based formulas, macaroni, spaghetti, HPLC, risk assessment

## Abstract

Aflatoxins are toxic secondary metabolites of fungal origin that contaminate agricultural commodities before, during and after harvest periods. A total of fifty-three (53) different foods (27 rice brands, 20 cereal based food brands and 6 pasta brands) were randomly obtained from the market and assessed for their different aflatoxin constitution (AFB1, AFB2, AFG1 and AFG2) as well as the total levels of the aflatoxins using High Performance Liquid Chromatography (HPLC) method. For the rice grain category, RS4 recorded the highest aflatoxin quantities of 65.77, 19.27, 1.02 μg/kg for AFB1, AFB2, AFG1 respectively and a total of 86.06 μg/kg which significantly differed (p < 0.05) from the other brands of foods. For the cereal based food category, CBS11 recorded the greatest quantities of 35.46, 4.92, 3.39 and 0.32 μg/kg for AFB1, AFB2, AFG1 and AFG2 respectively and a total of 45.1 μg/kg. For the pasta category, PS1 recorded the greatest quantities of 0.94 and 0.85 μg/kg for AFB1 and AFB2 respectively. Total aflatoxin quantities detected in some foods were above the acceptable limits set by the European Union which makes them unsafe and dangerous for human consumption. Recorded Estimated Daily Intakes (EDI) and Hazard Indices (HI) values were in the range of 3.9 × 10^-3^ – 0.899 and 3.9 × 10^-4^ - 0.0899 respectively. The risk was low since HI values obtained were less than 1.

## Highlights

• Majority 96.3% (26/27) of rice samples had total aflatoxin levels below international set limits (European Commission: 10 μg/kg).
• Almost half 45% (9/10) of the cereal based foods (mostly maize) were contaminated with aflatoxins and were above the set limits.
• None 0% (0/6) of the macaroni/spaghetti were contaminated with aflatoxins.
• AFG1 and AFG2 aflatoxin types were insignificant.

## 1 Introduction

Mycotoxins have become a major global concern and also, developed greatly during the last few decades due to their implications to human as well as animal health, productivity and trade. Several methods and strategies have been employed in addressing consumer concerns of various aspects of Food Safety [1] to regulate their occurrence. According to Jard et al. [2], more than 100 molds producing approximately 400 secondary metabolites of toxigenic potentials have been documented till date.

Fungi belonging to the genus *Aspergillus* are well noted for the production of aflatoxins which are a group of polyketide-derived furanocoumarin secondary metabolites. Species such as *A. flavus* (produces AFB1 and AFB2), *A. parasiticus* (produce AFB1, AFB2, AFG1 and AFG2*)*, *A. nomius* (produces AFB1, AFB2, AFG1 and AFG2), *A. ochraceoroseus* (produces AFB1, AFB2, AFG1 and AFG2 rarely), *A. pseudotamarii* (produces AFB1, AFB2, AFG1 and AFG2 rarely) and *Emericella venezuelensis* is a species with the perfect stage that produces AFB1, AFB2, AFG1 and AFG2 rarely has been implicated for the production of aflatoxins ([3,4, 5]). *Aspergillus bombycis* reported by Peterson et al. [6] isolated from insect frass in silkworm rearing houses in Japan has currently been added to the list as a new aflatoxigenic species.

With regards to aflatoxins, published findings of Inan et al. [7] suggested that there are a little over 20 recognized aflatoxins. Nonetheless, the four major ones critically considered are B1 (AFB1), aflatoxin B2 (AFB2), aflatoxin G1 (AFG1), and aflatoxin G2 (AFG2) while aflatoxin M1 (AFM1) and M2 (AFM2) are regarded as minor and have been reported as being the hydroxylated metabolites of AFB1 and AFB2 [8,9].

Among the five (5) notable mycotoxins in food, aflatoxins (B1, B2, G1 and G2) are the most toxic. Undoubtedly, aflatoxin B1 is the most studied as it has been identified as most toxic and potentially hepatocarcinogenic [10]. Ingestion of sufficient quantities for both livestock and humans, can be highly carcinogenic and acutely toxic or fatal [11] as their effects are realized through respiratory, mucous or cutaneous routes, ending in over activation of inflammatory response.

In Ghana and beyond, owing to their nutritional complementary significance and the quest to reduce malnutrition, cereals and legumes are by far the main ingredients for weaning foods as well as baby formulas ([12,13, 14]). Mycotoxin contamination of these foods consumed have been rampant and have not been spared [13].

The Food and Agricultural Organization (FAO) and World Health Organization (WHO) have also identified these toxins present in agricultural products and has resulted in the setting of maximum tolerable limits for mycotoxins in various countries [15]. According to the FDA regulatory levels for aflatoxin in the feed, the maximum allowable aflatoxin levels are 300, 100 and 20 μg/kg for finishing cattle, swine and poultry, breeding cattle, and other animals respectively [16].

The European Union in 2008, placed aflatoxin on the Rapid Alert System for Food and Feed (RASFF) due to its severe effects it caused (European [17]). Again, the International Agency for Research on Cancer (IARC) has also catalogued AFB1 as a group I carcinogen for humans [18,19]. Regrettably, their effect on health is seriously disregarded in developing countries including Ghana. Odamtten [20] reiterated that the danger of mycotoxicoses especially aflatoxicoses still exists in Africa and cannot be discounted in our health delivery system.

This paper sought to evaluate the risks and levels of the different types of aflatoxin as well as the total aflatoxin levels in some packaged rice, cereal based and pasta foods as sold on the markets of Ghana.

## 2 Materials and Methods

### 2.1 Sample collection

Samples were randomly purchased from the Makola market (Central market) in the period of May to July in 2015, Accra, Ghana and grouped into 3 categories (rice, cereal based food, pasta) (Table 1). Twenty (20) grams each of samples were kept in sterile bags and sent to laboratory where they were stored in a deep freezer at −20 °C until ready for chemical analysis.

**Table 1:** Coded products and their corresponding ingredients used in the experiment.

Product Codes Ingredients.
i) RS1-27 Rice
ii) CBS 1 Millet, soyabeans
iii) CBS 2 Maize
iv) CBS 3 Maize
v) CBS 4 Wheat, rice, millet, groundnuts, maize, soya
vi) CBS 5 Maize
vii) CBS 6 Maize
viii) CBS 7 Maize
ix) CBS 8 Maize
x) CBS 9 Maize
xi) CBS 10 Wheat, rice, millet, groundnuts, maize, soya beans
xii) CBS 11 Roast maize, soya beans, groundnuts
xiii) CBS 12 Roast maize, sorghum, groundnuts, soya beans
xiv) CBS 13 Maize, milk, additives
xv) CBS 14 Maize, sugar, dried mixed fruit flakes (apple, banana, strawberry) milk, barley
xvi) CBS 15 Wheat, honey, milk
xvii) CBS 16 Rice, salt, wheat, groundnuts, soyabeans
xviii) CBS 17 Maize, groundnuts, soyabeans
xix) CBS 18 Soyabeans, rice, groundnuts, wheat
xx) CBS 19 Maize, soyabeans, groundnuts
xxi) CBS 20 Maize, millet, wheat, groundnuts, soyabeans
xxii) PS1-6 Wheat

### 2.2 Preparation of samples for analysis

#### 2.2.1 Extraction of samples

AFB1, AFB2, AFG and AFG2 were extracted from samples according to the European Committee for Standardization (CEN) method for aflatoxin extraction [21]. Methanol in water (200 ml) (8 + 2) and 5 g NaCl were used to extract 25 g of sample. Hexane (100 ml) was added to samples containing more than 50% fat. The extracts were filtered and 10 ml of filtrate added to 60 ml of phosphate buffer for solid phase extraction using immune affinity columns specific for AFB1, AFB2, AFG1 and AFG2. This was followed by clean up with 15 ml distilled water and elution with methanol (HPLC grade). The eluate was injected into HPLC to estimate concentrations.

#### 2.2.2 HPLC parameters

Injection volume: 10 μl flow rate: 1 ml/min, column temperature: 35℃, excitation wavelength: 360 nm, emission wavelength: 440 nm, mobile phase composition: water/acetonitrile/MeOH (60/30/20 v/v/v), post column derivatization: Kobra cells.

HPLC Column Specification

Spherisorb ODS1- Excel

(4.6 mm x 25 cm), 5 μm particle size, 250 A pore size

LOD = Limit of detection

LOQ = Limit of quantification

CAN = Acetonitrile

MeOH = Methanol

LOD calculation= 3 x Baseline noise x Concentration

Peak Height

LOQ calculation = 2x LOD

Supplier of Column R- Biopharm, Block 10 campus, West Scotland Science Park, Acre road, Glasgow, Scotland G20 OXA.

2.3 Analysis of samples

**Fig. 1:**
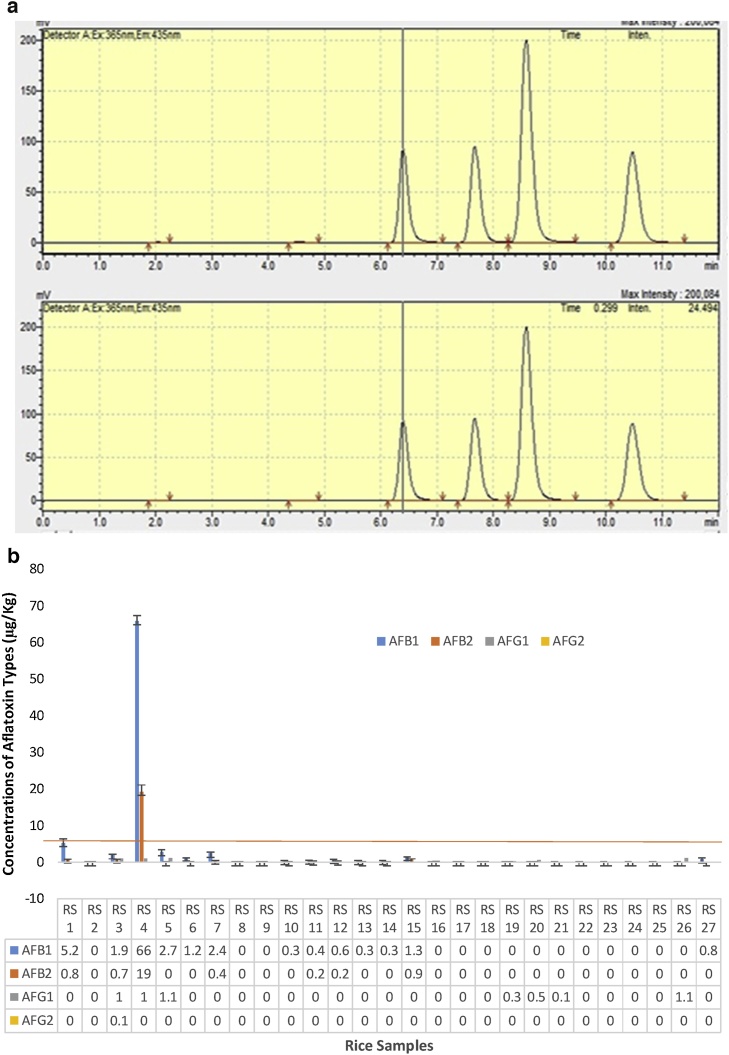
(a) Peak diagrams of the standards used in the experiment. (b) Concentrations of aflatoxin types of the different rice samples from Makola market (Central Accra), Ghana.

The aflatoxins (by *Aspergillus flavus and A. parasiticus*) levels in the samples were determined by High Performance Liquid Chromatography HPLC (Agilent 1260 Series, OpenLab software, X-bridge column (250 mm x 4.6 mm, i.d., 5 μm), USA with fluorescence detector and post column derivatization using kobra cells to generate bromine electrochemically at the CSIR- Food Research Institute, Ghana. LOD for AFB1 & AFB2 is 0.14 μg/kg and 0.13 μg/kg for AFG1 and AFG2 (Fig.1a).

### 2.3 Human risk assessment of exposure to total aflatoxins via consumption of rice, cereal based foods and pasta

#### 2.3.1 Exposure estimation

Calculation of the Estimated Daily Intake (EDI) was done by using the mean levels of aflatoxins obtained in rice, cereal based foods and pasta samples, the daily intakes of same samples [22,23], and the average body weight. The EDI for mean aflatoxins was calculated according to the following formula and expressed in μg/kg of bodyweight/day (μg/kg b.w/day) [24].

$$\text{EDI}\;=\;\frac{daily\;\mathrm{int}ake\;\left(\text{food}\right)\;\text{X}\;\text{mean}\;\text{level}\;\text{of}\;\text{Aflatoxins}}{\text{average}\;\text{body}\;\text{weight}}$$

#### 2.3.2 Estimation of Hazard Index (HI)

The Hazard Index (HI) was calculated according to the below mentioned formula, by dividing the EDI by TD_50_, divided by a safety factor of 50,000. TD_50_ is the dose (ng/kg/body weight/day) required to induce tumors in half of test animals that would have remained tumor-free at zero dose as described by Ishikawa et al., [25] and Ismail et al., [26].

$$HI\;=\sum_{\text{n}=0}^{\text{i}}\frac{\left(EDI/TD_{50}\right)}{5000}$$

### 2.4 Statistical Analysis

Regression analysis was used to calculate the concentrations of the aflatoxins from the curves derived from the standards of the aflatoxins with Excel for Microsoft windows (version 10). Means of results were subjected to analyses of variance (one-way ANOVA) at significant difference (p < 0.05) and separation of means were determined via post-hoc test using Duncan’s multiple range test DMRT with SPSS 16 (Chicago, USA). Means and standard deviations were computed and graphical representations were used appropriately.

## 3 Results

The samples and their corresponding ingredients investigated, have been described aptly in Table 1. Rice samples (RS1-27), varied combinations of cereals and other ingredients formed the cereal based foods (CBS 1-20) while pasta foods (PS1-6) composed mainly of wheat.

**Fig. 2:**
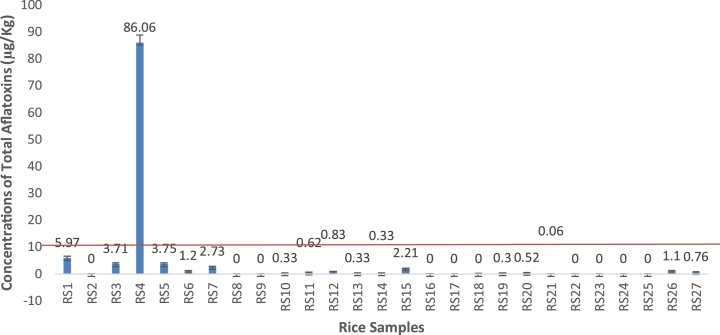
Total concentrations of aflatoxins of the different rice samples from Makola market (Central Accra), Ghana.

**Fig. 3:**
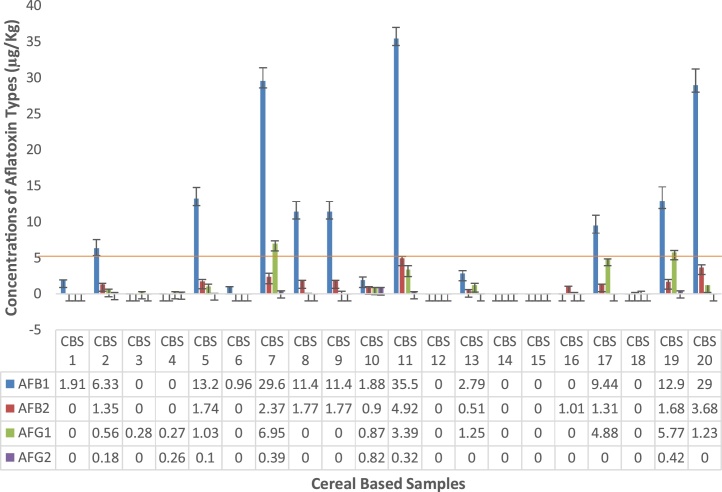
Concentrations of individual aflatoxin types in some cereal based foods from Makola market (Central Accra), Ghana.

**Fig. 4:**
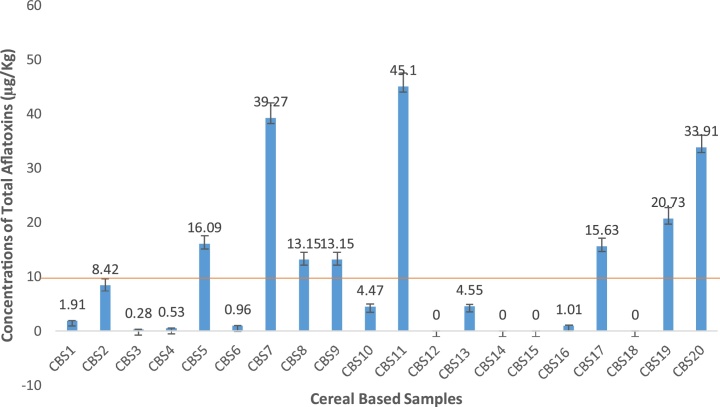
Total concentrations of aflatoxins in some cereal based foods from Makola market (Central Accra), Ghana.

**Fig. 5:**
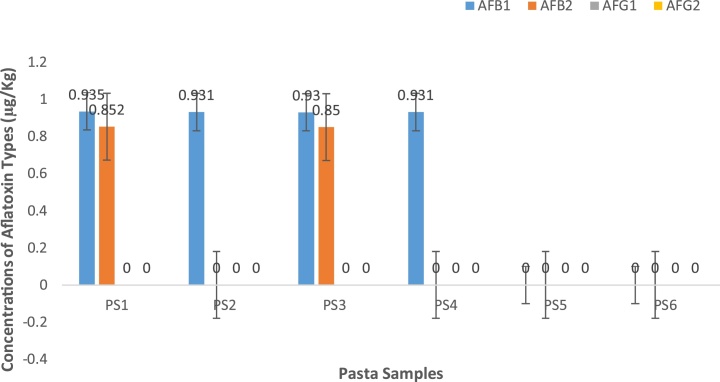
Concentrations of aflatoxin types in pasta foods from Makola market (Central Accra), Ghana.

**Fig. 6:**
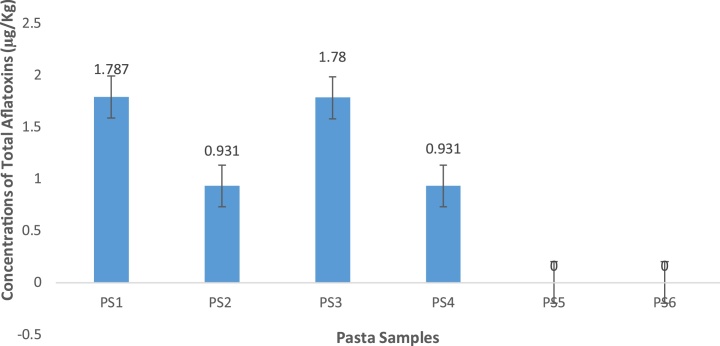
Total concentrations of aflatoxins in some pasta foods from Makola market (Central Accra), Ghana.

Results of the analyzed samples are presented in Fig. 1, Fig. 2, Fig. 3, Fig. 4, Fig. 5, Fig. 6. shows the type, occurrence and concentrations of the aflatoxins with respect to the types of samples (of rice, cereal based foods and pasta). A total of twenty-seven (27) different rice brands were selected randomly and analyzed. The order of levels was AFB1 > AFB2 > AFG1 > AFG2. According to Quinto et al., the critical point for determining the degree of biological activity of this group of fungal toxins is the terminal furan moiety of AFB1.

Aflatoxins type AFB1 levels for in the rice brands samples ranged from 65.77- Non detectable (N.D) levels. Value obtained for RS4 was significantly higher (p < 0.05) than all other sample brands. Moderate values were recorded in sample brands such as RS1 (5.2 μg/kg), RS5 (2.69 μg/kg), RS3 (1.87 μg/kg) and RS15 (1.33 μg/kg) which showed no significant difference (p > 0.05). The rest of the samples recorded low values of range 0.61 μg/kg to non- detectable levels.

Type AFB2 levels ranged from 19.27- 0.01 μg/kg (near non detectable)/Non- detectable. Again the sample of RS4 recorded significantly high (p < 0.05) values than the rest of the samples.

Type AFG1 and AFG2 yields recorded were very low and they ranged between 1.05- N.D (non- detectable) and 0.12- N.D (non- detectable) respectively. Statistically, there were no significant differences (p > 0.05) observed.

The Total aflatoxins yield was significantly (p < 0.05) highest in RS4 than all other sample brands. There was no significant (p > 0.05) difference observed among the other brands.

In the category of cereal based foods, a total of 20 foods were analyzed. Results of the analyzed samples are presented in Fig. 3, Fig. 4. Aflatoxins AFB1 levels ranged between 35.46- 0.96 μg/kg. and the rest were not detected. Values recorded for CBS11 (made from roasted maize, soyabeans and groundnut) was highest (p < 0.05) among all the samples. Following closely were CBS7 and CBS20 with values of 29.56 and 29.0 μg/kg respectively. There was no significant (p > 0.05) difference between them. However, they differed significantly from all the other samples analyzed.

Results for AFB2 ranged between 4.92-0.51 μg/kg while the rest were non detected. Again CBS11 recorded the highest value of 4.92 μg/kg and was statistically different (p < 0.05). Generally, there were statistical differences (p < 0.05) observed in all the samples analyzed.

AFG1 values recorded in this category was considerably high and ranged from 6.95-0.27 μg/kg. However, the rest were not detected. CBS7 recorded a significantly (p < 0.05) highest value of 6.95 μg/kg. Furthermore, CBS19, CBS17 and CBS11 recorded moderately high values of 5.77, 4.88 and 3.39 μg/kg respectively.

AFG2 values observed were generally low which ranged between 0.82-0.1 μg/kg. Statistically, there were no significant differences (p > 0.05) observed. AFG2 were not detected in the rest of the samples. Total aflatoxins levels recorded were within range of values of 45.1-0.28 μg/kg.

Results of the pasta category showed a total of six (6) different samples analyzed. For AFB1, quantities ranged between 0.930 to 0.935 μg/kg. There was no observed statistical difference (p > 0.05). AFB2 quantities ranged between 0.85 to 0.853 μg/kg. Again, there was no significant difference (p > 0.05). AFG1 and AFG2 levels were not detected in these samples.

Total aflatoxin levels for pasta samples ranged between 0.931 to 1.787 μg/kg. Interestingly, there was no significant difference (p > 0.05) observed between PS1 and PS3 both of which obtained the highest level of total aflatoxins. A similar trend was observed for PS2 and PS4. However, AFG1 and AFG2 were not detected in the six (6) different pasta food brands.

Daily intakes of foods obtained were of range 0.07-0.175 (adult), 0.47- 0.92 (infants) for rice, 0.12 (adults), 0.92 (infants) for cereal based foods and 0.175 (adults), 0.092 (infants) for pasta (Table 2).

**Table 2:** Daily intakes of the various food products in Ghana.

Food Sample	Daily Intake (Kg/person/day)	References
i) Rice	0.07- 0.175 (adults) 0.920 (infants)	Galbete et al [27], Regasa et al [28] Asare-Boadu and Syme [29] [30]
ii) Cereal based food	0.920 (infants) 0.267 (0.2-0.333) (6-8mths old) 0.40 (0.3-0.5) (9-11mths old) 0.73(0.55-0.917)(12-23mths old) 0.12 (adults)	[30] [31], Abeshu et al [32] Galbete et al. (2016)
iii) Pasta	0.175 (adults) 0.09 (infants)	Galbete et al. (2016)

Hazard index (HI)

Recorded HI values were in the range of 3.9 × 10^-4^ - 0.0899 (Table 3). In general, it is accepted that an HI ≤ 1 indicates no significant health risk. Nonetheless, the possibility of long-term adverse health effects increases with increasing HI values as an HI between 1.1 and 10 reflects a moderate risk, and HI < 10 indicates high risk [33]. HI value for exposure to aflatoxins via consumption of rice, cereal based foods and pasta consumed by the Ghanaian population, is less than one. These values this research is reporting imply that intake of rice, cereal based foods and pastas will most likely not pose high risk to the health of Ghanaian population.

**Table 3:** Estimated Daily Intake (EDI) and Hazard Indices (HI) for Ghanaians via consumption of rice, cereal based foods and pasta.

Food Sample	Mean total aflatoxins (μg/Kg)	Age	Average body wgt. (Kg)	Estimated Daily Intake(EDI) (μg/Kg.bw/day)	Hazard Index (HI)
i) Rice	6.52	18-60 yrs (adults)	60.7	0.013	1.3 × 10^-3^
		6-52 mths (infants)	7.0	0.111	0.0111
ii)Cereal based foods	13.69	18-60 yrs (adults)	60.7	0.271	0.0271
		6-52 mths (infants)	7.0	0.899	0.0899
iii) Pasta	1.36	18-60 yrs (adults)	60.7	3.9 × 10^-3^	3.9 × 10^-4^
		6-52 mths (infants)	7.0	0.017	1.7 × 10^-3^

TD50 = 0.2 ng/Kg.bw [68]

1 ng = 0.001 μg

Average body weight of an adult in Ghana = 60.7 Kg [69]

Average body weight of infants in Ghana = 7.0 (2.5-11.65) Kg ([67]; [32])

4 Discussion

In Ghana, the pervasiveness of *Aspergillus* species in stored dehydrated foods is a well-known phenomenon and has been well-documented [34]. In view of this, it is imperative to update the aflatoxicogenic potential of these fungi in relation to food commodities.

The rather surprising high levels of AFB1, AFB2 and total aflatoxin levels of recorded in RS4 and RS1 (Above EU limit of 5 and 10 μg/kg for AFB1 and total aflatoxins respectively) could be attributed to some favourable intrinsic factors such as pH, nutrient composition, moisture content/water activity as well as extrinsic factors as temperature; relative humidity; soil properties; mechanic injury on food commodity; insects and rodents attack [35,36]. Atanda et al. [35] also, suggested that these factors, however, do not work in solitude. Therefore, two or more factors may have to be met before fungal growth and corresponding toxin production can be effected.

Fungal infestation, growth and aflatoxin development is linked principally to water activity (Aw). This observation is attributable to improper drying which predisposes stored cereals and legumes to growth of mycotoxigenic fungi such as *Aspergillus* species which is conjectured to also increase with storage time [13,37,38]. The risk of *Aspergillus flavus* and its metabolites accumulation could be high at maturity and harvest of food crops at the end of the raining season. In Ghana, traditional drying techniques which comprise of field- and bare ground-drying; as well as poor handling contribute immensely to fungal contamination as the soil is known to be a reservoir of these fungi [39].

Recently, Zoreky et al., [5] reported values of 0.014- 0.123 μg/kg and 0.052-2.58 μg/kg for AFB1 and Total aflatoxins respectively in Saudi Arabia. In Nigeria, [40,41] reported values of 200.19 μg/kg and 37.9 μg/kg respectively as they investigated the aflatoxin levels in some human commodities. Makun et al. [42] also reported a value of 102.91984 mg/kg AFB1 in 27 out of 50 marketed wheat samples. Furthermore, in Pakistan, rice (Basmati) contained higher levels of both AFB1 and AFS. Their levels were 4.9–8.8, and 8.9–12.5 mg/kg, respectively as reported by Iqbal et al. [43].

Although the levels of aflatoxin contamination found in this study in rice for human consumption (up to 86.06 μg/kg) are far lower than the levels (1,600–12,000 μg/kg) reported by Afla-Guard [44], that caused deaths in the two fatal outbreaks of aflatoxin poisoning in Kenya accumulated intakes of small doses of aflatoxins may aggravate other clinical conditions such as immunosuppression, still-births and neonatal mortality with increased susceptibility to infectious diseases such as pneumonia, stunting of growth and HIV/AIDS [45,46]. It is indeed of great concern as protracted intakes of such toxic levels could similarly work synergestically with other carcinogens especially hepatitis B virus to aggravate primary liver cancer [47]. It is noteworthy that no exposure to any level of aflatoxins could be regarded as safe [48].

Results of the maize samples agreed with published findings of Sirma et al [49] whom from a study conducted in Kenya, showed that 60% of total household maize samples (269) across the study tested positive for aflatoxin with a range of 0.17-5.3 ppb while all 39 millet samples recorded aflatoxin levels ranging from 0.14 - 6.4 ppb, none of which exceeded the country's regulatory limit of 10 ppb. In another study, 55% of maize products in Kenya reported by Lewis et al [50] had aflatoxin levels greater than the set limit of 20 ppb, while 35% of the products had levels exceeding 100 μg/kg and7% above 1000 μg/kg. From Malawi, a range of 1.47- 22.47 μg/kg was reported by Mwalwayo and Thole [51] in maize samples consumed in the rural areas.

Recently, Omeiza et al [52] also reported a range of 1.8- 13.5 μg/kg in feed samples in Nigeria

On the contrary, sorghum samples recorded aflatoxin levels ranging from 0.15 to 210.1 ppb (overall mean of 26.0 ppb), out of which 46% of samples (from the Kilibwoni sub-location) exceeding the maximum tolerable limit of 10 ppb registered the highest percentage which suggests a possible variation in aflatoxin frequency with geographical location

The results by Sugri et al. [53] for aflatoxin prevalence range of 0.011-308 ppb in maize samples (240) in six districts in the Upper East and Upper West regions of Ghana. Kumi et al. [54] from Ghana, reported aflatoxin levels in homemade weanimix ranging from 7.9 –500 ppb. The data obtained from their study showed that two (2) out of the thirty-six (36) samples (from different communities) had very high levels of 460 and 500 ppb (ug/kg). Aflatoxin contamination in maize grains from a total of 38 major store markets in Benin, Ghana and Togo was monitored by James et al. [55] and reported aflatoxin concentration in contaminated samples of range 24 - 117.5 ng/g,0.4 -490.6 ng/g, and 0.7- 108.8 ng/g in Benin, Ghana and Togo respectively.

Pasta samples results obtained agreed with findings of Raiola et al. [56] who reported range of values from non-detection limits to very low values for aflatoxins in pasta from Italy. Our results contradicted published findings of Cagindi [57] who reported values of total aflatoxin in the samples of range 0.04 to 10.20 μg/kg of an average of 5.52 μg/kg in mesir pasta in Turkey. From Pakistan, Iqbal et al. [58] reported macaroni (5.91 μg/kg), noodles (7.35 μg/kg) and spaghetti (9.12 μg/kg). Ezekiel and Sombie [59] also reported 1.1, 2.2 and 1.2 ppb for macaroni, noodles and spaghetti respectively in Nigeria. The incidence of acute hepatic encephalopathy in children, had been linked to aflatoxin contamination of noodles with an approximate 3 mg suspected to be present in a single serving in Malaysia [60]

It has to be underscored that majority 96.3% (26/27) of the rice samples had total aflatoxin levels below acceptable limits (10 μg/kg) set by the 77 countries that regulate aflatoxins, including the European Union ([61]; EC 2006). However, approximately half 45% (9/20) of cereal based foods had AFB1 and total aflatoxins within the acceptable levels stipulated by the EU regulations for AFB1 and total aflatoxins in rice. The pasta category of samples had all samples within acceptable limits set by the EU.

Interestingly, majority of the samples had very low to no value for AFG1 likewise AFG2 (Figs. 1– 3). In this standing, it was reported by some previous researchers ([62]; [5]) that AFB1 is considered to be a precursor of AFG1 and thus explaining the relative accumulation of both toxins. It was also reported by Reiter et al., [3], that cross reactivity by AFG1 to the AFB1 antibodies occur at higher percentages in immune ultra-filtration cleanup of aflatoxins.

The danger for the consumer who may purchase and use an opened bag over a long period and sometimes even beyond the expiry date, may expose himself to contaminated products since these produce is often kept in moist/damp storage houses for a very long time. The health implications are enormous in Africa since most produce are not properly dried.

Results obtained in this research for EDI (3.9 × 10^-3^ -0.899 μg/Kg.bw/day) were higher compared to values reported by Oyedele et al [63] (91.2 ng/Kg. bw/day) from Nigeria. Kooprasertying et al., [64] also reported high aflatoxin concentrations of peanuts sold in Thailand but at lower exposure risks (0.36–1.91 ngg^−1^).

## 4 Conclusion

It can be surmised from our results that samples had 3.7% (1/27), 45% (9/20) and no aflatoxins levels respectively for rice, maize based foods and spaghetti with aflatoxin above permitted levels (E.U) (≤ 5 and 10 μg/kg for AFB1 and total aflatoxins respectively). The Hazard Indices recorded values less than 1 and so Ghanaians consuming these products were not at risk.

This evaluation of mycotoxins in Ghanaian rice gives an indication of the quality of the cereal with regards to its acceptability for human and animal consumption. The demonstrated presence of AFs at concentrations above the limits acceptable to world mycotoxin regulatory agencies and the co-occurrences of toxins with possible toxic synergistic effects make this studied rice samples of low quality for human and animal consumption and in fact raises preliminarily national public health concerns. However, with appropriate measures such as awareness creation and proper management practices, the prevalence of the toxin can be minimized, if not eradicated.

## 5 Authors’ contributions

NKK, AAA and FA performed the experiments and wrote the manuscript. VK-B was responsible for AFB1, AFB2, AFG1 and AFG2 analysis. NKK, AAA, HWA, FA and VK-B helped conceive the experiments and prepared the manuscript. NKK, AAA and FA conceived the original study and HWA and VK-B led the sampling and study in Ghana. All authors read and approved the final manuscript.

## Conflicts of interest

The authors declare that there are no conflicts of interest.

## Transparency document

Transparency document
